# A randomized trial evaluating an mHealth system to monitor and enhance adherence to pharmacotherapy for alcohol use disorders

**DOI:** 10.1186/1940-0640-7-9

**Published:** 2012-06-08

**Authors:** Susan A Stoner, Christian S Hendershot

**Affiliations:** 1 Talaria, Inc, 1121 34th Ave, Seattle, WA, 98122, USA; 2 Department of Anesthesiology and Pain Medicine, University of Washington, 1959 NE Pacific Street, BB-1469, Seattle, WA, 98195, USA; 3 Centre for Addiction and Mental Health, 33 Russell Street, Toronto, ON, M5S 2S1, Canada; 4 Department of Psychiatry, University of Toronto, 250 College Street, Toronto, ON, M5T 1R8, Canada; 5 The Mind Research Network, 1101 Yale Boulevard NE, Albuquerque, NM, 87106, USA

**Keywords:** Alcohol dependence, Medication adherence, mHealth, Internet intervention, Opioid antagonist

## Abstract

**Background:**

Nonadherence to prescribed medication regimens is a substantial barrier to the pharmacological management of alcohol use disorders. The availability of low-cost, sustainable interventions that maximize medication adherence would likely lead to improved treatment outcomes. Mobile health (mHealth) technologies are increasingly being adopted as a method of delivering behavioral health interventions and represent a promising tool for adherence interventions. We are evaluating a cell-phone–based intervention called AGATE that seeks to enhance adherence with regular text-messaging.

**Methods/Design:**

A randomized controlled effectiveness trial in the context of an eight-week open label naltrexone efficacy trial delivered in a naturalistic clinical setting. Treatment-seeking heavy drinkers (N = 105) are currently being recruited and randomly assigned to the AGATE intervention or a control condition. Daily measures of alcohol use and medication side effects are being recorded via cell phone in both conditions. Additionally, participants randomized to the AGATE condition receive medication reminders via SMS text message according to a schedule that adjusts according to their level of adherence.

**Discussion:**

Results from this trial will provide initial information about the feasibility and efficacy of mHealth interventions for improving adherence to alcohol pharmacotherapies.

**Trial Registration:**

NCT01349985.

## Background

Alcohol-related disorders contribute significantly to the global disease burden, accounting for an estimated 3.8% of deaths and 4.6% of disability-adjusted life years worldwide [1]. In the United States, recent estimates of annual economic costs attributed to alcohol consumption surpass $220 billon, equivalent to 2–3% of the gross domestic product [1,2]. Pharmacotherapy is one efficacious treatment option [3-5]; however, underutilization of pharmacotherapy and medication nonadherence remain significant barriers to its clinical utility [3,6-8]. Alcohol use itself is a significant risk factor for nonadherence to medication regimens [9-11], further undermining the potential efficacy of pharmacotherapy for alcohol problems.

Naltrexone is one of three medications approved by the US Food and Drug Administration for the treatment of alcohol dependence. Naltrexone has shown efficacy for reducing levels of alcohol use in those diagnosed with alcohol dependence [12] and may also facilitate reductions in heavy drinking in nondependent individuals with high-risk drinking [13]. Satisfactory adherence to naltrexone is critical to its efficacy, as demonstrated in several clinical trials [14-18]. Unfortunately, average rates of adherence to naltrexone pharmacotherapy are suboptimal, ranging from 55–85% over short-term periods (e.g., 12–16 weeks) [14,16-21] with poorer rates observed over lengthier assessment periods [20]. These estimates are based mainly on clinical trials that often include regular monitoring and are, therefore, likely to overestimate rates of adherence that can be expected in naturalistic settings. Naltrexone remains relatively underutilized by US physicians, who cite concerns about nonadherence as the most common reason for not prescribing this potentially valuable treatment [8]. Thus, identification and remediation of barriers to naltrexone adherence in naturalistic settings could help address prescribers’ concerns about nonadherence and improve clinical outcomes.

Ongoing monitoring of medication adherence is likely critical for maximizing treatment efficacy. A recent systematic review of randomized placebo-controlled studies of naltrexone for alcohol problems reported a significant association between the intensity of adherence assessments and the likelihood of returning to heavy drinking during treatment [7]. Although structured behavioral programs are available to help maximize medication adherence during pharmacological management of alcohol use disorders [22], the implementation of these programs in naturalistic settings is variable at best. In addition, interventions to improve adherence to alcohol pharmacotherapies often assess adherence on, at most, a weekly basis [22]. Consequently, there is a need for supplemental interventions to maximize adherence that are easily implemented, sustainable, and capable of reaching participants on a regular (if not daily) basis.

Mobile health (mHealth) technologies are increasingly being used to deliver behavioral health interventions [23] with one application being the delivery of mobile interventions for improving medication adherence [24,25]. The current randomized trial was designed to evaluate the efficacy of an mHealth intervention to improve adherence to naltrexone among treatment-seeking individuals with alcohol dependence or at-risk drinking. This cell-phone–based intervention consists of an adaptive goal-directed adherence tracking and enhancement (AGATE) system for measuring and enhancing medication adherence.

## Methods/Design

### Overview of AGATE

The AGATE system was developed at Talaria, Inc. (Seattle, WA). To summarize, AGATE uses a combination of Short Message Service (SMS) text messages and internet access on a cellular handset to conduct medication adherence assessments and reminders. On many handsets (e.g., Windows Mobile, iPhone, Blackberry), SMS messages permit the inclusion of live hyperlinks that can launch web-based assessments. All major carriers in the US and abroad make it possible for cellular subscribers to receive SMS messages via email by providing email addresses for this purpose. Thus, knowledge of the patient’s cell phone number and carrier are sufficient to deliver SMS messages containing assessments and medication reminders.

An important feature of AGATE is its adaptive capabilities based on self-reported adherence patterns. The program was designed so that the patient and provider agree upon, or the researcher sets, an adherence goal (e.g., a minimum of 90% of scheduled doses to be taken over a certain period of time, such as 14 days). In the first stage, patients or research participants receive a reminder/assessment message for every scheduled dose. If, at the end of this period, the patient has achieved the goal, he or she progresses to Stage 2, in which the reminder/assessment frequency steps down (e.g., to once every few days) for another period of time, such as 21 days. At the end of this second period, if the goal is maintained, the patient progresses to Stage 3, in which the reminder/assessment frequency steps down further (e.g., to once per week). However, if the goal is *not* maintained in Stage 2 or 3, messaging frequency steps back up, and the patient returns to the preceding stage. In this context, patients are provided regular feedback on their performance and are reminded of their goal. Participants can also be asked to complete daily assessments of drinking behavior, such as number of standard drinks per day and urge to drink, using the AGATE system.

### Study setting and overview

The purpose of this trial (ClinicalTrials.gov identifier NCT01349985) is to evaluate whether AGATE effectively measures and enhances medication adherence in the context of a brief naltrexone trial involving treatment-seeking heavy drinkers. Currently underway at the Mind Research Network (Albuquerque, NM), this open-label trial seeks to enroll up to 105 treatment-seeking heavy drinkers from the area. Qualifying participants must be interested in either reducing or stopping their drinking and deemed to be appropriate candidates for naltrexone by a physician. Those enrolled in the study will be prescribed naltrexone (50 mg once daily) for eight weeks. To simulate a real-world treatment scenario to the extent possible, participants will be assessed at an outpatient psychiatric clinic and will meet with a physician for baseline and follow-up visits at four and eight weeks after initiating medication.

All procedures were reviewed and approved by the University of New Mexico Health Sciences Center Human Research Review Committee (HRRC), which retains primary oversight, as well as Quorum Review (Seattle, WA).

All participants will receive smart phones during the study. Participants will be randomly assigned to receive either AGATE (adherence support) or a Structured Alcohol and Side Effects Diary (SASED) via smart phone. The primary outcome will be percent of scheduled doses taken during the eight week trial as measured by electronic pill cap monitoring (Medication Event Monitoring System [MEMS], Aardex Group, Union City, CA). Secondary outcomes will include other indices of medication adherence assessed via MEMS as well as those measured via pharmacist pill counts and self-reported adherence using the Timeline Followback (TLFB) method [26]. Tertiary outcomes will be selected indicators of alcohol use over the eight-week trial.

For the current study, SMS reminders to take medication will be tailored based on the participant’s level of adherence to that point. At the outset, participants will be reminded daily to take their medication. Upon achieving adherence for a period of several days, reminders will be reduced to once every third day. Participants in the control SASED condition will not receive any medication reminders; however, all participants will complete daily drinking measures (e.g., number of standard drinks per day, urge to drink) via the smart phone interface. These assessments will be sent each morning to assess drinking and craving during the prior day and will be identical across treatment conditions. Additionally, possible side effects will be assessed daily using brief assessments delivered concurrent with the drinking assessments. Finally, we will collect online metrics of number of messages ignored versus answered and latency to respond to messages to gauge the use and uptake of both AGATE and SASED.

### Participants and recruitment

Participants will be men and women aged 21–55 years who report average weekly consumption over the past 3 months of ≥ 14 standard drinks for women and ≥ 21 standard drinks for men assessed via phone screening and confirmed using TLFB [26]. Additionally, participants must a) report at least two heavy drinking days (defined as 4+ drinks in a day for women and 5+ drinks in a day for men) in a consecutive 30-day period within the three months prior to baseline evaluation [12]; b) endorse a desire to reduce or stop drinking alcohol and be willing to try naltrexone; and c) receive medical clearance by the study physician to take naltrexone based on blood panels and a medical exam.

Exclusion criteria include clinically significant physical illness; pregnancy or intent to become pregnant in the forthcoming 8 weeks; current (30-day) drug use other than alcohol, nicotine, or cannabis as verified by urine toxicology screen; any recent (past 90-day) use of opioids or other medications; lifetime opioid dependence [27]; current psychiatric medication other than antidepressants; lifetime diagnosis of a DSM-IV Axis I psychotic disorder [27]; baseline score ≥8 on the Clinical Institute Withdrawal Assessment for Alcohol scale [28] (indicating clinically significant alcohol withdrawal); liver enzyme levels greater than three times the upper limit of the normal range; concurrent receipt of another pharmacological or behavioral alcohol treatment (with the exception of Alcoholics Anonymous); or current court mandate (or other requirement) to attend treatment for alcohol problems.

### Procedures

Potential participants who meet basic eligibility criteria during preliminary phone screening will be scheduled for an in-person screening visit. At the in-person screening visit, participants will meet with the study coordinator to review the details of the study and provide informed consent. They will then complete the Structured Clinical Interview for DSM-IV-TR Axis I Disorders (SCID) [27] and a TLFB assessment of drinking behavior over the preceding 30 days [26] to confirm presence of inclusion criteria and absence of exclusion criteria. Alcohol dependence will be assessed using the SCID but is not a requirement for inclusion. Those meeting eligibility criteria will provide blood samples for hepatic tests and a urine sample for a toxicology screen. Additionally, female participants will complete a urine-based pregnancy screen. Pending no contraindications based on blood or urine screen results (which will be available within 2–3 days after the screening visit), participants will be scheduled for their baseline (Week 0) appointment. At that time, participants will be asked to remain abstinent from alcohol for 72 hours prior to their appointment to permit an assessment of potential withdrawal. At the baseline visit, participants will meet with the study physician and study coordinator to further determine whether naltrexone is medically appropriate based on additional medical and psychiatric criteria and to be assessed for withdrawal symptoms.

If no other medical or psychiatric contraindications are noted, the participant’s naltrexone prescription will be forwarded to the pharmacy, and he or she will proceed with the baseline session by completing a battery of study questionnaires administered by computer. Following completion of these questionnaires, the participant will meet with the study coordinator for detailed instructions on using the cell phone and responding to prompts delivered by the cell phone. At this point, the participant’s ID number will be entered into the web-based AGATE/SASED administrative interface, where computerized randomization will occur. This system is maintained by Talaria, Inc., but accessible to the study’s research assistant via web interface using a secure password. Participants will then be asked to select a drinking goal of either abstinence or moderation (the latter to be defined by the participant). The computer randomization algorithm will block participants according to drinking goal (abstinence or moderation), ensuring even distribution of these variables between conditions.

The study coordinator will provide each participant with a smart phone (Samsung® Prevail) and instruct the participant in its use. This overview will include basic functions of the phone, how to respond to SMS prompts, and guidelines for acceptable use of the phone during the study. Finally, the participant will pick up his or her four-week prescription for naltrexone from the on-site pharmacy. The pharmacist will fit the pill containers with the MEMS monitoring system, which will record an electronic timestamp each time the container is opened (these data are stored in a microchip in the MEMS cap).

Following the baseline visit, the course of the medication trial will be eight weeks. During this time, participants will return for follow-up medical visits at weeks four and eight, consistent with recommended clinical guidelines for monitoring patients receiving naltrexone [19]. At the visit, participants will meet with the study physician and provide a blood sample. They will also bring their medication bottles for pill counts and uploading of MEMS cap data, and will report whether they have had any problems with their phone or the AGATE system. Participants will then complete a brief subset of the study questionnaires (e.g., assessment of drinking and medication adherence in the last four weeks). If any medical contraindications to continuing naltrexone are in evidence, treatment will be discontinued. If no contraindications to continuing naltrexone are in evidence, the next four-week prescription will be sent to the pharmacy for participant pick-up. Vials will be refitted with a MEMS monitoring system by the pharmacist. At week eight, the study physician will complete a visit identical to week four (i.e., it will include the same questionnaires, pill counts, blood draw, and medical visit). However, study participation will terminate following the week-eight visit. Participants will be provided with a discharge recommendation from the study physician and a referral for continuing treatment if requested by the participant. At the end of the eight-week visit, the study coordinator will conduct an exit interview to elicit qualitative information on the usability and acceptability of AGATE/SASED.

### Power and sample size considerations

The primary outcome will be percent of scheduled doses taken during the eight-week trial as measured by MEMS. We will dichotomize participants according to whether they achieved the adherence criterion of 80%.

Examining antiretroviral medication adherence, Simoni *et al.*[29] compared an electronic pager adherence support intervention, also developed by Talaria, with a peer adherence support intervention and usual care (i.e., no adherence support). In that study, both interventions lasted three months; however, pages gradually tapered in the third month. Participants were characterized as either 100% adherent in the preceding week or not based on self-report and MEMS. At week two, self-reports indicated that 100% adherence was achieved by 80.4% in the pager arm, 63.2% in the peer-support arm, and 64.9% in the usual-care arm. This represents a medium effect size for the pager intervention compared with peer intervention (*w* = 0.36) and usual care (*w* = 0.32). At month three, after pages had been tapered, self-reports indicated that 100% adherence was achieved by 57.1% in the pager arm, 56.1% in the peer-support arm, and 47.4% in the usual-care arm. This represents no effect for the pager intervention compared with the peer intervention (*w* = 0.02), and a small-to-medium effect size for the pager intervention compared with usual care (*w* = 0.19). It is important to note that the pagers used in the Simoni *et al.* study functioned only as a high-technology reminder system. The pager did not assess and reinforce adherence as AGATE will. Moreover, the pages tapered without regard to adherence performance. Therefore, we anticipate a medium effect size for AGATE compared with SASED (*w* = 0.30) over the two-month course of the evaluation. Power analysis will be conducted using G*Power 3 software [30,31]. For a *χ*2 test with α error probability set to 0.05, a sample size of 88 will yield power of 0.80 to detect effect size *w* = 0.30. Allowing for up to 15–20% attrition between randomization and discharge, our target number of randomized participants is 105.

### Data analysis plan

Preliminary analyses will identify data input errors, outliers, and other threats to the integrity of subsequent analyses. Screening and baseline variables will be examined to determine whether randomization effectively produced equivalent groups. Any variable that is not equivalent between groups at screening or baseline will be used as a covariable in subsequent analyses as appropriate. To test our primary hypothesis that those in the AGATE condition will be more likely to be adherent over the course of the study, we will dichotomize the adherence outcomes based on MEMS assessments. Those who have taken ≥ 80% of scheduled doses will be classified as adherent; all others will be classified as nonadherent. The *χ*2 goodness-of-fit test will be conducted. To test our secondary hypothesis, that those in the AGATE condition will take significantly more scheduled doses, we will conduct multivariable analysis of variance with repeated measures. The dependent variables will be the AGATE, pill counts, MEMS, and TLFB measures of adherence. To test our tertiary hypothesis, that those in the AGATE condition will have better drinking outcomes, we will conduct multivariable analysis of variance with repeated measures. Percentage of days abstinent, percentage of heavy drinking days, drinks per drinking day, and latency to first heavy drinking day will be computed from the TLFB and from AGATE/SASED daily drinking reports and serve as dependent variables for this analysis. We will also examine concordance among AGATE, pill count, MEMS, and TLFB measures of adherence, globally and over time, using bivariable Spearman rank-order correlations (ρ). Degree of concordance will be tested with the κ statistic, which is corrected for chance association, and we will test for differences in adherence rates among the adherence measures using nonparametric Friedman two-way analysis of variance.

### Rationale for non-inclusion of a placebo condition

The present study has three primary purposes: to evaluate the effectiveness of AGATE as an adherence-enhancement intervention, to evaluate the validity and reliability of AGATE as an adherence-measurement system, and to evaluate the feasibility and acceptability of AGATE in the context of a trial of naltrexone treatment for problem drinking. The effectiveness of naltrexone relative to placebo is already established in the literature [32-34]. Because nonadherence to naltrexone is also well-documented in the literature [6,14,17,18,21,35-37] and adherence to naltrexone appears worse than adherence to placebo, presumably due to side effects [38], we felt that evaluating AGATE as an adherence-enhancement intervention in an open-label trial without placebo was sufficient for this efficacy study. Specifically, we are interested in knowing whether AGATE can help overcome reasons for nonadherence that go hand-in-hand with receipt of an active drug versus with placebo (e.g., certain mild drug-induced side effects) as well as reasons for nonadherence that would be expected in both placebo and active drug conditions (e.g., uncertainty regarding medication effectiveness, lack of understanding of the need for consistent dosing, waning motivation to reduce drinking, perceived stigma, reluctance to mix medications with alcohol or drugs [38]). Therefore, we wanted to maximize the number of participants receiving the active drug.

### Rationale for the selected control condition (SASED)

An obvious choice of a possible control condition could have consisted of adding a medication management (MM) component as was done in the Combining Medications and Behavioral Interventions for Alcoholism (COMBINE) study [12,37]. However, due to the additional time spent attending to compliance issues in an MM component, this method would create differential clinician attention between conditions. Furthermore, if participants in one condition received a smart phone with voice and internet service while the others did not, one could argue that a differential demand characteristic would be created between conditions. Because differential clinician attention and demand characteristics can create confounds, we reasoned that the most well-controlled and rigorous test of AGATE would be to create a control condition in which participants also receive a smart phone.

Researchers have previously used electronic diaries successfully to measure drinking on a daily basis [39] or episodically in an ecological momentary assessment paradigm [40]. For example, Bernhardt *et al.*[39] compared daily self-reports of alcohol consumption collected using a handheld computer with those collected using the TLFB method [26] among college students. Both methods enabled computation of overall alcohol consumption, drinks per drinking day, and number of heavy drinking days. No significant differences between the two methods were found. Thus, we felt that a good use of smart phones in the control condition would be to assess alcohol consumption on a daily basis. We also felt another good use of smart phones in the control condition would be to assess side effects on a weekly basis. Therefore, the control condition for the proposed study is a smart-phone alcohol and side-effects diary (SASED).

## Discussion

Results from prior randomized trials indicate that optimal adherence to naltrexone is critical to achieving medication-related reductions in drinking [7,16-18]. In light of these findings, the fact that naltrexone shows only modest efficacy across studies [28] could be partly reflective of suboptimal procedures for assuring good adherence in many clinical trials [7]. Thus, implementing behavioral interventions that improve adherence could increase medication effect sizes in clinical trials as well as improve treatment outcomes in real-world settings [7]. Promising behavioral interventions for increasing medication adherence in people with problem drinking have been developed and implemented successfully in clinical trials (22). Nonetheless, the fact that these interventions require ongoing assessment and intervention from clinicians is likely to limit uptake in naturalistic settings. Conversely, the AGATE intervention can be delivered on an ongoing basis at minimal cost. Moreover, because medication-taking is a behavior that must be enacted daily, adherence interventions should ideally be capable of reaching the individual on a frequent basis. Mobile health interventions are well suited to this objective.

Results from the current trial will inform further development of the AGATE system, which is currently being generalized to apply to any medication type or regimen. If results of this trial indicate the AGATE system is effective for improving medication adherence among treatment-seeking individuals with alcohol dependence or at-risk drinking, future studies will evaluate whether it can be effective in other contexts, including other naturalistic settings. Additionally, user feedback collected during this trial will be used to tailor subsequent iterations of the technology. A future goal is to make AGATE fully customizable for various medication schedules and to enable broad use for clinicians and researchers.

## Competing interests

SS declares that she is a full-time, salaried employee of Talaria, Inc., the company that developed the AGATE system. She holds no equity stake in the company. CH declares that he has no competing interests.

## Authors’ contributions

SS and CH both contributed to writing this manuscript and reviewed and approved the final draft.
